# Bootstrap Signal-to-Noise Confidence Intervals: An Objective Method for Subject Exclusion and Quality Control in ERP Studies

**DOI:** 10.3389/fnhum.2016.00050

**Published:** 2016-02-12

**Authors:** Nathan A. Parks, Matthew A. Gannon, Stephanie M. Long, Madeleine E. Young

**Affiliations:** Department of Psychological Science, University of ArkansasFayetteville, AR, USA

**Keywords:** event-related potentials (ERP), signal-to-noise ratio (SNR), bootstrap confidence intervals, subject exclusion criteria

## Abstract

Analysis of event-related potential (ERP) data includes several steps to ensure that ERPs meet an appropriate level of signal quality. One such step, subject exclusion, rejects subject data if ERP waveforms fail to meet an appropriate level of signal quality. Subject exclusion is an important quality control step in the ERP analysis pipeline as it ensures that statistical inference is based only upon those subjects exhibiting clear evoked brain responses. This critical quality control step is most often performed simply through visual inspection of subject-level ERPs by investigators. Such an approach is qualitative, subjective, and susceptible to investigator bias, as there are no standards as to what constitutes an ERP of sufficient signal quality. Here, we describe a standardized and objective method for quantifying waveform quality in individual subjects and establishing criteria for subject exclusion. The approach uses bootstrap resampling of ERP waveforms (from a pool of all available trials) to compute a signal-to-noise ratio confidence interval (SNR-CI) for individual subject waveforms. The lower bound of this SNR-CI (*SNR_LB_*) yields an effective and objective measure of signal quality as it ensures that ERP waveforms statistically exceed a desired signal-to-noise criterion. *SNR_LB_* provides a quantifiable metric of individual subject ERP quality and eliminates the need for subjective evaluation of waveform quality by the investigator. We detail the SNR-CI methodology, establish the efficacy of employing this approach with Monte Carlo simulations, and demonstrate its utility in practice when applied to ERP datasets.

## Introduction

Analysis of data in event-related potentials (ERP) studies includes two major quality control steps that exclude data from further analysis: *artifact rejection* and *subject exclusion*. Artifact rejection occurs at the level of individual trials, removing segments of electroencephalogram (EEG) containing non-brain signals (artifacts) from inclusion in waveform averages. This step ensures that mean ERP waveforms are not grossly contaminated by non-brain signals such as muscle activity, eye movements, impedance fluctuations, or amplifier blocking. There are numerous methods in the ERP literature to quantify, detect, and reject/correct non-brain artifacts which use methods of minimum/maximum voltage criteria, spectral decomposition (Goncharova et al., 2003; Delorme et al., 2007), and independent component analysis (ICA; Jung et al., 2000; Joyce et al., 2004; Delorme et al., 2007). Few analytic options exist for the second layer of data removal for ERP quality control, subject exclusion. The step of subject exclusion discards individual subject ERPs from grand mean waveforms and group-level statistics if these waveforms fail to meet a sufficient level of evoked signal quality. That is, subject exclusion is used to remove subjects whose waveforms fail to clearly emerge over baseline noise levels. A wide variety of factors can impact the quality of waveforms at the subject level: low trial counts, excessive artifact, shifts in electrode impedance, failure to fixate attention/vigilance, fatigue/exhaustion, task disengagement. Because many of these factors (and the interactions between them) are not easily detected, documented, or quantified, subject exclusion on the basis of waveform quality is generally a necessary and critical step of quality control in ERP experiments as it ensures that each subject included in an analysis exhibits a stable evoked brain response. However, unlike the step of artifact rejection, the ERP literature lacks quantitative approaches to subject exclusion and current methods are qualitative, subjective, and lack standardization.

To elucidate the issues of subject exclusion in the ERP literature, we conducted a review of the Method sections of ERP papers published over 3 years (2012–2014) in two neuroscience journals that publish a high proportion of ERP papers (*Neuroimage* and *Psychophysiology*). Out of a total of 331 ERP papers published from 2012 through 2014, 45.9% (152 of 331) reported excluding subjects on the basis of electrophysiological signals. Of those papers excluding subjects, 69.1% (105 of 152) did not report any quantifiable criteria and provided only qualitative justifications for excluding subjects on the basis of their ERP signal quality (e.g., descriptors such as “low signal-to-noise”). Thus, the most common approach for evaluating the quality of individual subject data appears to be the visual inspection of ERP waveforms by investigators. Such an approach is problematic, as there are no established criteria of what constitutes a “good” waveform nor do individual subject waveforms adhere to the canonical patterns that emerge in grand average waveforms. Without clear and objective standards, decision criteria for subject exclusion are likely to vary considerably across laboratories, investigators, and experiments. Furthermore, visual inspection of ERPs is most commonly conducted on mean waveforms of individual conditions or the grand mean of all conditions. The former may introduce biases in selecting subjects for exclusion whereas the latter misrepresents the signal-to-noise ratio (SNR) of individual experimental conditions (formed from far fewer trials).

A second issue was also apparent from our review of ERP Method sections. Though a number of papers did report numeric criteria to justify subject exclusion (30.9%; 47 of 152), these criteria were always based upon an arbitrary number or percentage of trials available for averaging following artifact rejection procedures. Though the proportion of trials used in ERP signal averaging is certainly related to the resultant waveform’s quality, this relationship is poorly defined at the subject level. That is, there is no single value that can accommodate the broad range of individual differences in ERP signal-to-noise. For example, 50% of artifact-free trials may produce a strong ERP waveform in one subject whereas 100% may yield a low quality waveform in another. Moreover, there was no standardization of numeric criteria in the ERP literature. Thresholds established for subject exclusion varied considerably from paper to paper, ranging from 15 to 75% of rejected trials. Thus, even when numeric criteria are used, the selected threshold is arbitrary and has a poorly established correspondence to signal quality.

A final notable point from our review of ERP subject exclusion procedures is that the majority of ERP papers (54.1%; 179 of 331) did not report excluding any subjects on the basis of ERP data quality. However, this does not imply that all subjects included in these analyses had high quality ERP waveforms. As discussed above, passing current criterion for subject exclusion provides no assurance of signal quality as there are no established or standardized methods for doing so. Furthermore, the absence of subject exclusion reporting does not necessarily imply that all subjects should have been included in analyses. An explicit statement that all subjects passed rejection criteria and exhibited high quality ERPs was given in only 3 of 179 papers. As such, the other 176 papers either did not report that all subjects had passed criteria, did not perform a subject exclusion step, or only reported post-exclusion sample sizes. Thus, even in papers that have not excluded subjects, the quality of individual ERPs in the sample cannot be assured.

The issues of subject exclusion in the ERP literature described above clearly highlight a great need for an objective, quantitative, and standardized approach for subject exclusion and data quality assurance in ERP experiments. Here, we describe such a method: a simple approach that uses bootstrap resampling to compute a SNR confidence interval (SNR-CI) for individual subject ERP waveforms. Bootstrap resampling methods have been used with wide success in subject-level ERP analyses to obtain measures of reliability (Fabiani et al., 1998; Fortune et al., 2004), detect the presence of ERP components (Lv et al., 2007; McCubbin et al., 2008), and perform hypothesis testing (Di Nocera and Ferlazzo, 2000; Oruç et al., 2011). The method described here quantifies the signal strength of an ERP waveform as SNR (expressed in dB) and uses a bootstrap resampling procedure (Efron, 1979; Efron and Tibshirani, 1994) to compute an SNR-CI. The lower bound of this SNR-CI, the *SNR_LB_*, yields a value of SNR that a subject’s waveform has statistically exceeded. Thus, *SNR_LB_* quantifies ERP signal quality as a statistical boundary of a waveform’s SNR. *SNR_LB_* can be evaluated against a desired criterion to objectively exclude individual subjects from group-level analyses. Summary statistics of *SNR_LB_* can also serve, more generally, as a metric of ERP signal quality which can be reported in ERP manuscripts to convey the quality of a sample at the subject level. We describe this bootstrap SNR-CI approach in detail, perform a set of Monte Carlo simulations to demonstrate its efficacy, and then apply it to an existing ERP dataset to demonstrate its utility as criterion for subject and metric of signal quality. The code for computing bootstrap estimates of *SNR_LB_* is freely available at http://www.uark.edu/ua/parkslab/SNRLB and https://figshare.com/s/f6da4150953b0f9cc3bd.

## Bootstrapped Erp Snr-Cis

The signal processing logic of ERPs is that by averaging numerous segments of event-locked EEG, overlapping but uncorrelated sources of noise will average out to reveal an underlying phase-locked waveform. Thus, the most basic feature defining an ERP waveform is that, relative to a pre-stimulus baseline, the post-stimulus interval exhibits significant voltage deflections relative to zero. The strength of such post-stimulus voltage deflections can be readily quantified as the SNR of the post-stimulus interval relative to the pre-stimulus baseline. SNR for a mean ERP waveform^1^ can be derived from the formula:

(1) SNRERP=20 log10(RMSPOSTRMSPRE)

where, *RMS_POST_* is the square root of the mean squared (root mean square, or RMS) of the voltage within a time window of interest, *RMS_PRE_* is the RMS of the ERP pre-stimulus baseline, and *SNR_ERP_* is the resultant SNR for the time window of interest, expressed logarithmically in decibels (dB).

*SNR_ERP_* provides a simple and straightforward measure that quantifies the signal strength of an ERP waveform. However, a single point estimate of SNR derived from a mean ERP does not fully portray the quality of an evoked signal as it does not capture the variability of the signal. A CI of SNR forms a more appropriate metric of ERP signal quality as it provides a measure of the signal’s reliability. That is, the lower bound of the SNR-CI (*SNR_LB_*) can be interpreted as the level of SNR that is statistically exceeded by the waveform. Thus, *SNR_LB_* provides a measure of assurance that a desired SNR criterion has been met. For example, a subject’s ERP could have a mean SNR of 10 dB but a CI of [−1 dB, 21 dB]. Though mean SNR is relatively high, this subject’s waveform should be considered unacceptable as it fails to statistically exceed even 0 dB, a value of SNR indicating no more signal than noise.

**Figure 1:**
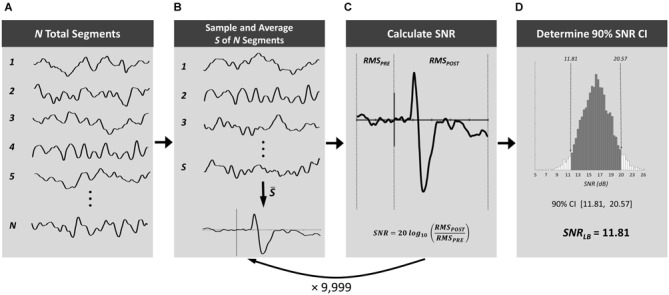
**Procedure for computing bootstrap SNR-CIs**. From the set of all electroencephalogram (EEG) segments from a subject **(A)**, a number of segments equivalent to that going into ERP condition means, *S*, are randomly sampled with replacement and signal averaged **(B)**. SNR is then calculated for the time window of interest **(C)**. This process is repeated 9999 times to obtain a large distribution of SNR values and obtain a 90% CI **(D)**.

Our SNR-CI method employs bootstrap resampling of ERPs to compute *SNR_LB_* for individual subject waveforms by randomly drawing from an aggregate pool of EEG segments to obtain a distribution of SNR values and derive CIs (Figure 1). The pool of EEG segments is the set of all segments (*N*) that will be used to form mean ERP waveforms (Figure 1A). Thus, *N* is the set of EEG segments prior to their categorization into specific experimental conditions but subsequent to preprocessing (e.g., filtering, ocular correction) and artifact rejection. From this set of *N* segments, *S* segments are randomly sampled (with replacement), signal averaged, and baseline corrected to form a bootstrap ERP waveform (Figure 1B). The number of segments sampled, *S*, should be equivalent to the number of segments that will be used to calculate mean ERP waveforms in each experimental condition. For example, if an experiment had 800 total trials (surviving artifact rejection) that were divided equally among eight experimental conditions, the value of *S* would be 100. *SNR_ERP_* is then derived from each resampled bootstrap ERP according to formula [1] (Figure 1C). Bootstrapping of ERPs and SNR is repeated 9999 times to obtain a large distribution of SNR values, based on resampled ERPs derived from all experimental conditions. *SNR_LB_* is then derived as the lower bound of a 90% CI of this distribution of 9999 SNR values (Figure 1D).

## Monte Carlo Simulations of *SNR_LB_* Performance

**Figure 2:**
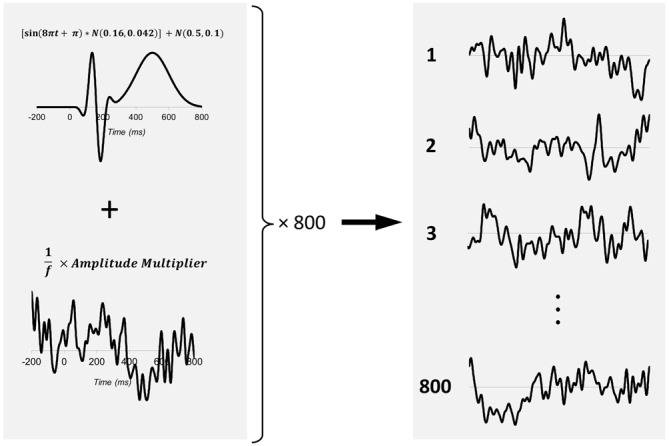
**Method of generating synthetic ERP subjects**. For each subject, 800 EEG segments (−200 to 800 ms) are generated. These segments consist of an artificially generated canonical waveform (top left) consisting of an 8 Hz one-dimensional Gabor summed with a Gaussian according to the formula: [sin(8Π*t* + Π) × *N* (0.16, 0.042)] + *N* (0.5, 0.1) This canonical waveform was then summed with an equivalent length of 1/f noise. The noise level of each synthetic subject was manipulated by scaling the amplitude of 1/f noise via a noise multiplier factor that varied between 5 and 35 times.

We performed a set of Monte Carlo simulations to examine the utility of employing SNR-CIs as a metric of ERP signal quality and criterion for subject exclusion. These simulations generated large datasets of synthetic ERP subjects, each of which was composed of 800 segments containing a prototypical ERP waveform embedded within varying levels of pink noise (1/f noise). We generated a waveform to approximate a canonical ERP waveform by summating an 8 Hz one-dimensional Gabor with a Gaussian (Figure 2). All waveform components peaked at ±1 (arbitrary units) and had latencies and frequency approximating those of P1, N1, and P3 components of the visual evoked potential (the most commonly measured class of ERP). An equivalent interval (−200 to 800 ms) of 1/f noise was then generated, normalized, low-pass filtered at 30 Hz, and summated with the ERP waveform. Eight hundred such segments were generated for each simulated ERP subject. The noise level within each simulated subject was manipulated by multiplying the amplitude of 1/f noise by a factor varying randomly between 5.0 and 35.0 prior to its summation with the ERP waveform. Manipulating 1/f amplitude in this way allowed samples of synthetic subjects with wide ranges of SNR to be generated.

### Optimal Number of Bootstraps for Computing *SNR_LB_*

**Figure 3:**
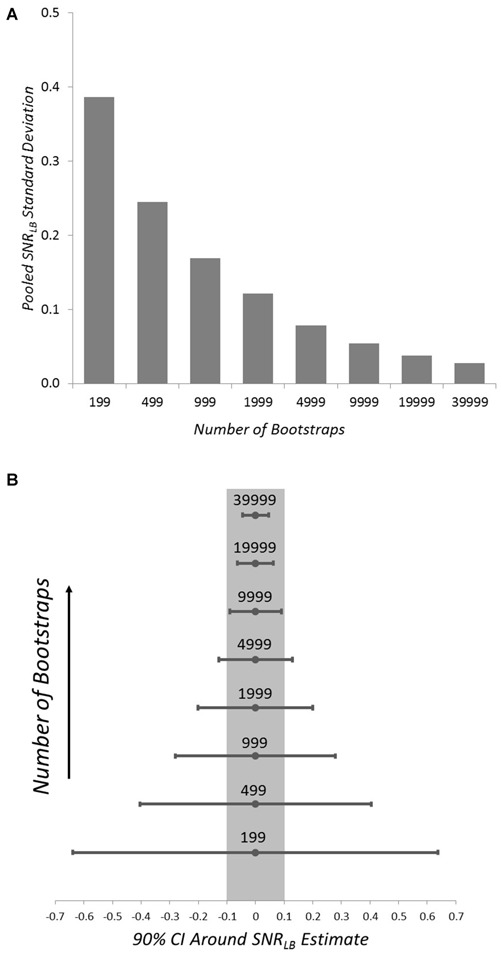
**Pooled SD (A) and 90% CIs (B) of *SNR_LB_* error for the tested bootstrap values in 100 synthetic ERP subjects**. The grayed region in **(B)** indicates those bootstrap values below the targeted 90% CI error ± 0.1 dB.

Because of the rather demanding computational resources required to perform bootstrap resampling of ERP waveforms, we first sought to determine the minimum number of bootstrap iterations that could be used to compute *SNR_LB_* without significant impact on the error of *SNR_LB_* estimates. To this end, we evaluated the minimum number of bootstraps needed to yield a confidence interval of less than ±0.1 dB in *SNR_LB_* estimates. To determine the number of bootstraps required to stay within this margin of error, we generated 100 synthetic ERP subjects. For each of these subjects *SNR_LB_* was measured 30 times at each of eight bootstrap values: 199, 499, 999, 1999, 4999, 9999, 19,999, and 39,999. We then obtained the *SNR_LB_* SD for each bootstrap value within each synthetic subject. SD values were then pooled across all 100 subjects for each bootstrap value (Figure 3A). From these pooled SDs, we derived 90% CIs for each bootstrap value (Figure 3B). A value of 9999 bootstraps yielded a CI of ±0.09 dB around the *SNR_LB_* estimate. This value of 9999 bootstraps was employed in all subsequent simulations and applications of *SNR_LB_*.

### Classifying the Presence of an ERP with *SNR_LB_*

**Figure 4:**
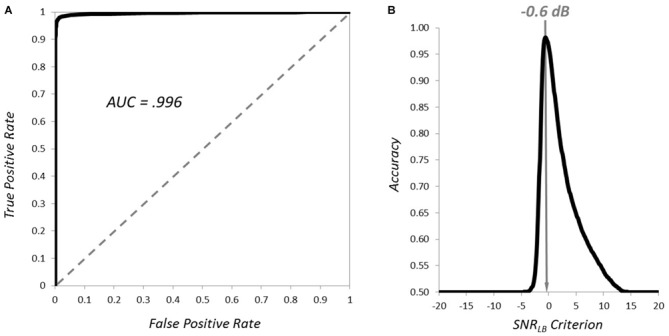
**Classification of the presence and absence of ERP waveforms by *SNR_LB_***. The receiver operating characteristic (ROC) curve for classification of signal present vs. signal absent synthetic subjects is plotted in **(A)** and the mean classification accuracy of *SNR_LB_* at criterion levels from −20 to +20 dB is plotted in **(B)**.

We next sought to demonstrate that *SNR_LB_* provided an effective criterion for simply classifying the presence (vs. absence) of an ERP waveform embedded in varying levels of noise. We generated 5000 synthetic subjects that contained an underlying ERP (signal present) and 5000 with segments consisting entirely of 1/f noise (signal absent). *SNR_LB_* was then computed for each subject by resampling 200 segments 9999 times (*N* = 800; *S* = 200; 9999 bootstraps). The accuracy of *SNR_LB_* at classifying subjects as signal present vs. signal absent was then evaluated at 401 criterion ranging between −20 dB and +20 dB (0.1 dB increments). The resultant receiver operating characteristic (ROC) curve is plotted in Figure 4A. Area under the ROC curve was 0.996, indicating exceptional accuracy at classifying signal present vs. signal absent subjects. Overall classification accuracy (mean of true and false positives) was best (>90%) at criterion levels between −1.2 and 0.9 dB, with peak classification accuracy of 98.2% at −0.6 dB (Figure 4B). These simulations clearly demonstrate that *SNR_LB_* can serve as an effective metric for establishing the presence of an underlying ERP signal embedded within varying levels of noise. However, it should be noted, that although a criterion of −0.6 dB yielded the greatest accuracy in classifying the presence of a signal, this criterion value is too low in practice. Any value of *SNR_LB_* less than or equal to 0 dB is much too liberal as it indicates that the ERP waveform does not reliably exceed baseline levels of noise.

### Classifying the Quality of an ERP with *SNR_LB_*

**Figure 5:**
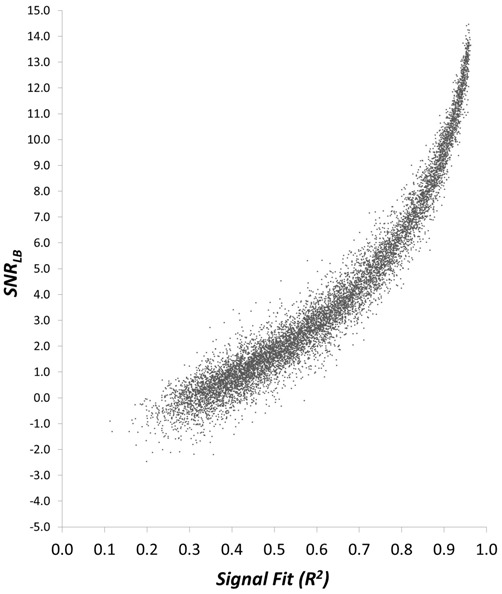
**Scatterplot of the relationship between *SNR_LB_* and the statistical fit (*R*^2^) of a synthetic subject to the underlying ERP waveform**.

The above simulation demonstrates that *SNR_LB_* is an accurate metric to classify the presence vs. absence of an underlying waveform and provides proof of concept that *SNR_LB_* can be utilized as a criterion for ERP subject exclusion. However, merely classifying the presence vs. absence of a signal is an oversimplification of the subject exclusion process. The problem of excluding ERP subjects is not to make a simple determination as to whether or not a signal is present. Rather, the problem is to determine whether or not an evoked response has achieved a sufficient level of signal quality. To assess *SNR_LB_* as such a metric we first examined the relationship between *SNR_LB_* and the quality of an underlying ERP waveform. Because the true underlying waveform is known in our simulated datasets, the statistical fit (*R*^2^) between the ERP of a synthetic subject and the actual underlying signal can be calculated. The obtained value of *R*^2^ then provides a quantitative index of signal quality that can be evaluated against *SNR_LB_*. We generated a sample of 10,000 synthetic subjects, each containing an underlying ERP waveform embedded in a randomly selected noise level (random noise multiplier between 5.0 and 35.0; Figure 2). For each synthetic subject’s dataset, we computed a bootstrap estimate of *SNR_LB_* (*N* = 800, *S* = 200, 9999 bootstraps). For each of these 9999 bootstraps we also calculated an *R*^2^ between each bootstrap ERP and the underlying waveform, then obtained a mean *R*^2^ value for each subject. We then correlated these *R*^2^ values with *SNR_LB_* using Spearman’s rho (*r_s_*). There was an exceptionally strong monotonic relationship between ERP signal fit and *SNR_LB_* (*r_s_* = 0.973, *p* < 0.0001, *R*^2^ = 0.947), where increasing values of *R^2^* were associated with increasing estimates of *SNR_LB_* (Figure 5). This strong correlation indicates that *SNR_LB_* can serve as a proxy measure of ERP signal quality.

**Figure 6:**
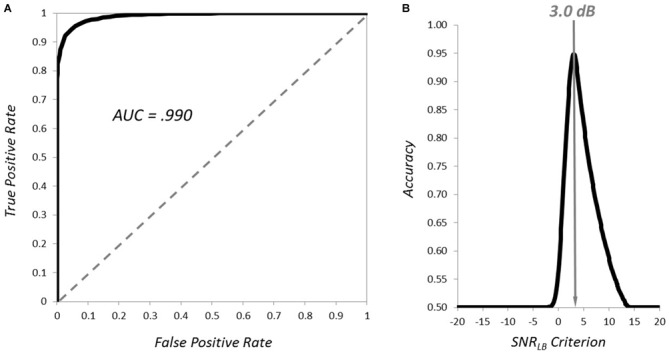
**Classification of the quality of ERP waveforms by *SNR_LB_***. The ROC curve for classification of passing vs. failing quality subjects is plotted in **(A)** and the mean classification accuracy at criterion levels from −20 to +20 dB is plotted in **(B)**.

Next, we examined how well *SNR_LB_* could classify subject waveforms according to signal quality. More specifically, we defined a threshold level of signal fit (*R*^2^) and examined the performance of *SNR_LB_* in correctly classifying synthetic subject waveforms as “passing” (≥*R*^2^ threshold) or “failing” (<*R*^2^ threshold). To determine an appropriate threshold value for this *R*^2^ threshold we reasoned that, at a minimum, for an ERP to be considered of sufficient quality, it must achieve a better fit to the true underlying signal than waveforms at the statistical boundary of signal and noise. As noted above, an *SNR_LB_* value of zero can be considered such a boundary. As such, we define the minimum “passing” quality ERP waveforms as those that achieve significantly better statistical fits to the underlying signal than waveforms with *SNR_LB_* of zero. To determine a threshold *R*^2^ value, we generated a sample (5000 simulated subjects) with a mean *SNR_LB_* of zero. We identified an appropriate range of noise to achieve such a sample by generating small samples of 100 subjects at 10 levels of the 1/f noise multiplier, identifying the approximate zero crossing, reducing the scale and repeating the process until a sufficient level of accuracy (±0.05 dB) was achieved. This process yielded to a noise range of 36.0 ± 0.25 dB (normally distributed). We then generated 5000 subjects within this range of 1/f noise. For each of these synthetic subjects, we computed *SNR_LB_* (*N* = 800, *S* = 200, 9999 bootstraps) and mean values of *R*^2^ (the mean fit of the subject’s bootstrap ERPs to the underlying waveform). This yielded a distribution of both *SNR_LB_* and *R*^2^ for the 5000 synthetic subjects. The mean of the *SNR_LB_* distribution was 0.001 dB with a 90% CI of [−1.244, 1.224], yielding a distribution of 5000 subjects with a mean *SNR_LB_* approximating zero. The *R*^2^ distribution had a mean of 0.3111 and a 90% CI of [0.2063, 0.4172]. The upper bound of this *R*^2^ distribution (0.4172) then provides a threshold *R*^2^ value to determine “passing” and “failing” levels of ERP signal quality. We then categorized each subject from the previous sample of 10,000 (noise levels between 5.0 and 35.0) as passing or failing based upon 90% CI of each synthetic subject’s bootstrap *R*^2^ distribution. Subjects were classified as passing if the lower bound of their *R*^2^ CI exceeded the threshold value of 0.4172 (4078 subjects) and were categorized as failing if it did not (5282 subjects). The accuracy of *SNR_LB_* at classifying subjects as having passing or failing quality was evaluated at 401 criterion ranging between −20 dB and +20 dB (0.1 dB increments). The ROC curve and overall classification accuracy is plotted in Figure 6. Area under the ROC curve was 0.990, indicating exceptional accuracy at classifying subjects according to signal quality (Figure 6A). Mean classification accuracy was best (>90%) at criterion levels between 2.3 and 3.9 dB, with peak classification accuracy of 94.82% at 3.0 dB (Figure 6B). These simulations suggest that *SNR_LB_* can serve as an efficient classifier of signal quality and that an *SNR_LB_* criterion value of 3.0 dB should serve as a minimum threshold for the inclusion of subjects in ERP experiments.

**Figure 7:**
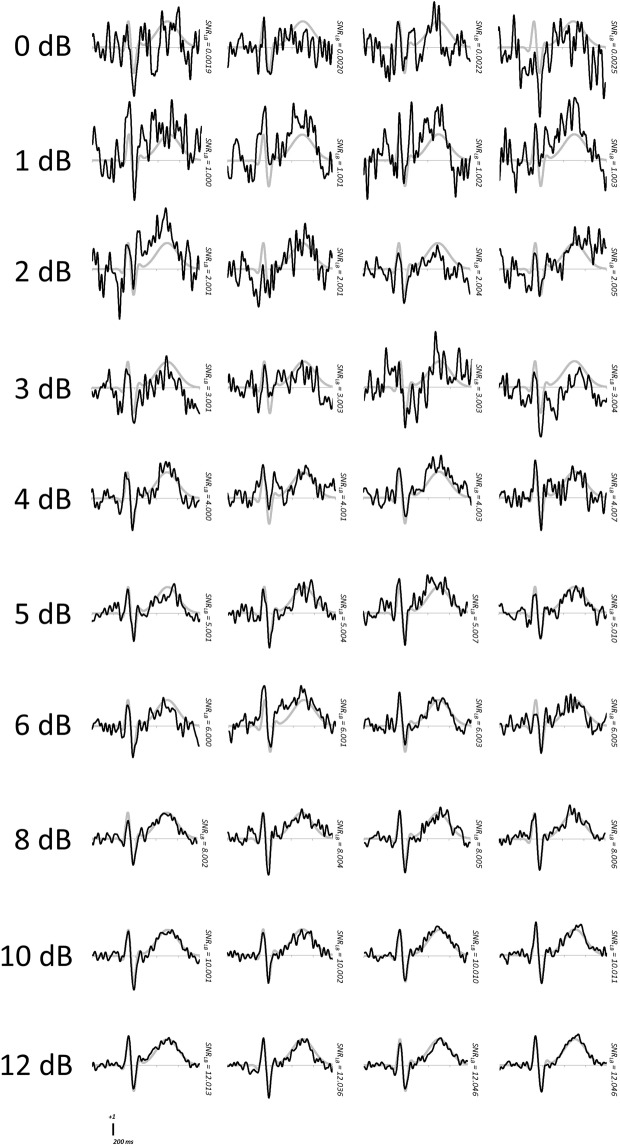
**Representative waveforms of synthetic subjects achieving increasing criteria of *SNR_LB_***.The waveforms of the first four subjects to achieve or surpass a given criterion level are plotted in black. The true underlying waveform is plotted in gray.

To further demonstrate the relationship between *SNR_LB_* and ERP waveform quality, synthetic subject waveforms are plotted in Figure 7. These waveforms represent the resampled ERP at the median bootstrap SNR value. Out of the 10,000 subjects in the generated sample, those shown are the first four to surpass *SNR_LB_* thresholds of 0, 1, 2, 3, 4, 5, 6, 8, 10, and 12 dB. These plots provide visual confirmation that ERP signal quality improves with increasing values of SNR*_LB_*. These plots further illustrate that waveforms with values of *SNR_LB_* at or below 3.0 dB would generally be considered excessively noisy when visually inspected by ERP investigators.

The simulations described here demonstrate a strong relationship between *SNR_LB_* and ERP signal quality (as indexed by statistical fit) and clearly demonstrate the efficacy of *SNR_LB_* in classifying ERP waveforms according to signal quality. Simulations further suggest an *SNR_LB_* value of 3.0 dB to serve as a minimum threshold of signal quality, establishing a standardized metric and guideline for subject exclusion in ERP studies.

## Manipulating *SNR_LB_* in individual Subject Erps

The above Monte Carlo simulations indicate that *SNR_LB_* can serve as a quantifiable criterion for subject exclusion and effective metric of ERP signal quality. To further demonstrate the utility of using *SNR_LB_* and the appropriateness of establishing a minimum threshold value of 3.0 dB, we provide a qualitative demonstration of the relationship between *SNR_LB_* and visual ERP signal quality in a real ERP dataset. To this end, we manipulated the SNR of individual subject waveforms in a small ERP dataset to qualitatively evaluate the appearance and pattern of ERP waveforms with increasing values of *SNR_LB_*. We provide such a demonstration by manipulating levels of noise within individual subjects in two ways. First, we combined EEG segments time-locked to a stimulus onset with segments drawn from arbitrary time periods in the ongoing EEG. Second, we simply manipulated the number of trials, *S*, used to derive bootstrap ERPs.

We collected EEG from four subjects as they completed 1000 trials of a visual oddball paradigm. Three of these four subjects are authors on this paper (MG, SL, and MY). The fourth subject was naïve as to the purposes of this study. Every 1000–1500 ms, a sinusoidal grating flashed for 200 ms. The orientation of the flashed grating was probabilistically determined, being vertical with a probability of 0.8 and horizontal with a probability of 0.2. Subjects counted the number of horizontal gratings (oddball). EEG was recorded from 64 scalp-record channels using a BrainAmp DC configured with the ActiCap active electrode system (Brain Products, Munich, Germany). At acquisition, data were recorded in reference to electrode FCz and sampled at 1000 Hz (DC to 250 Hz). Data were analyzed offline using BrainVision Analyzer (Brain Products, Munich, Germany). EEG data were re-referenced to the average of the left and right mastoids, ocular corrected (Gratton et al., 1983), band-pass filtered 0.1–30 Hz (zero phase-shift Butterworth, 24 dB/octave), and segmented −200 to 800 ms relative to stimulus onset. We also derived segments of EEG from arbitrary inter-stimulus time points, to obtain a set of EEG segments that contained no underlying ERP signal (“noise” segments). All EEG segments were then baseline corrected according to the prestimulus interval and rejected as artifacts if voltage exceeded ±150 μV. Remaining segments were then pooled across electrodes O1/2, P7/8, PO7/8, PO3/4, P1/2, and PO3/4.

**Figure 8:**
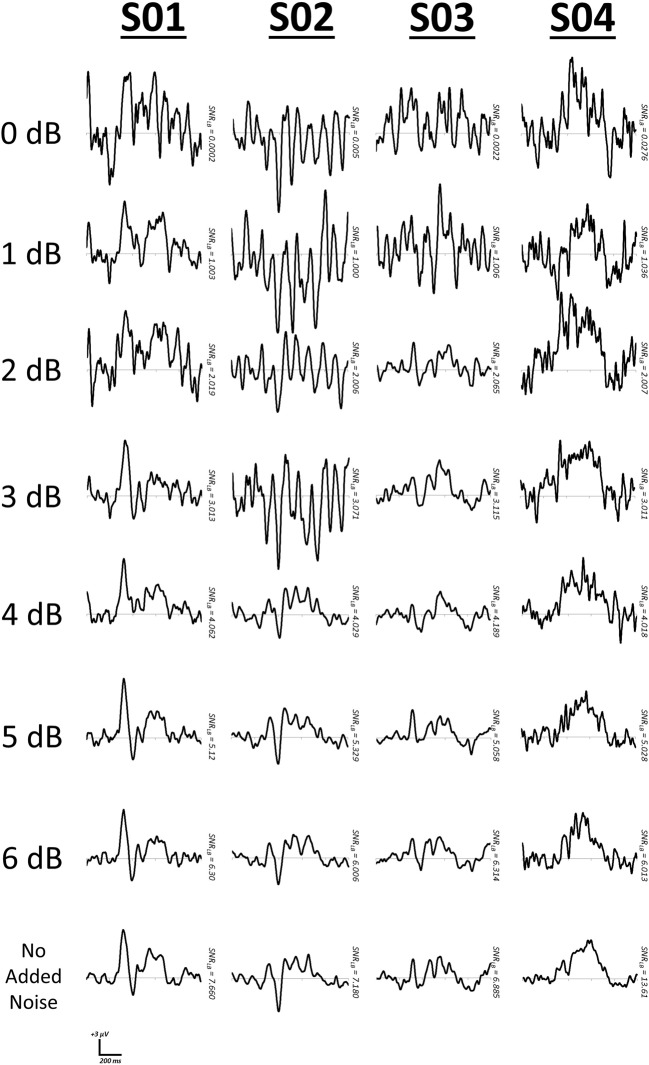
**Representative waveforms at increasing criteria of *SNR_LB_***. SNR was manipulated by mixing EEG segments time-locked to a stimulus with segments derived from arbitrary time points.

First, we manipulated *SNR_LB_* within each of the four subjects by summating varying proportions of signal present EEG segments with signal-absent segments. That is, for all segments available for a subject, signal-absent segments would be added in varying proportions to signal-present segments. For example, a level of 0.5 indicates that 50% of the available segments were summed with arbitrary EEG segments, whereas a level of 1.0 indicates that all signal-present segments were summed with arbitrary segments. Using this approach we iterated the proportion of arbitrary EEG segments (increments of 0.05) and computed bootstrap estimates of *SNR_LB_* (*S* = 200, 9999 bootstraps). We then determined those values at which *SNR_LB_* first surpassed thresholds of 0, 1, 2, 3, 4, 5, and 6 dB^2^. Representative bootstrap waveforms were extracted at the median SNR value (Figure 8).

**Figure 9:**
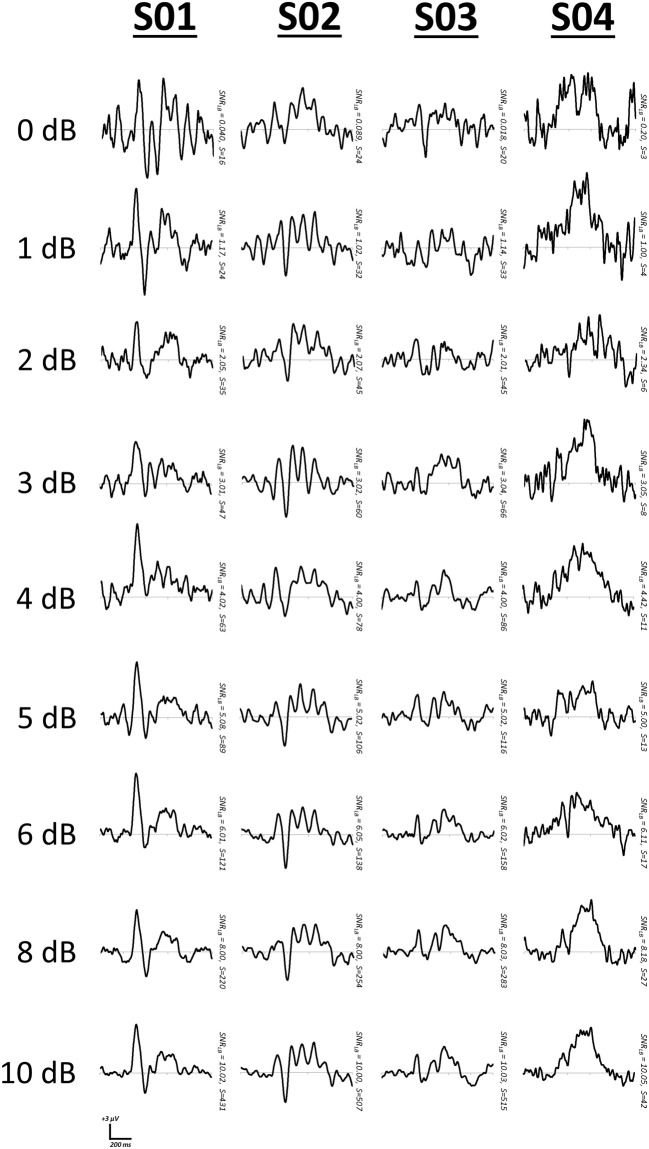
**Representative waveforms at increasing criteria of *SNR_LB_***. SNR in each subject was manipulated by controlling the number of segments, *S*, used to form signal averages during the bootstrap SNR-CI computation.

We also manipulated SNR in these four subjects using a second method of simply varying the number of EEG segments used to derive bootstrap ERPs. That is, we iterated the value of *S* in the bootstrap SNR procedure (Figure 1B) from 2 to 525. We then determined the values of *S* at which SNR_LB_ first surpassed thresholds between 0 and 10 dB^3^ (1.0 dB increments). After determining the values of *S* at which these SNR lower bound criteria were achieved, representative bootstrap ERP waveforms were extracted at median SNR values. Figure 9 plots representative ERP waveforms in each of the four subjects as the specified SNR lower bound criteria were met.

As with simulated waveforms, manipulation of SNR in real EEG datasets from individual subjects also demonstrates a clear relationship between *SNR_LB_* and ERP signal quality. As *SNR_LB_* surpasses the suggested minimum threshold of 3.0 dB, ERPs begin to increasingly conform to clearer and more stable waveform patterns (Figures 8, 9).

## *SNR_LB_* Application: Excluding Subjects in An Erp Dataset

As a final qualitative illustration of the utility of *SNR_LB_* as a metric of subject exclusion in ERP datasets, we applied the SNR-CI method to an existing ERP dataset to demonstrate the quality of ERP subject waveforms that fail to pass the recommended *SNR_LB_* criterion of 3.0 dB.

We applied SNR-CIs to an ERP dataset derived from an experiment investigating the impact of visual category learning on visual evoked potentials (VEPs). EEG data were collected from 33 subjects recruited from the University of Arkansas undergraduate population. All procedures were approved by the University of Arkansas Institutional Review Board. EEG data were recorded using a 64-channel BrainAmp ActiCap active electrode system (Brain Products, Munich, Germany). EEG were recorded in reference to electrode FCz, digitized at 1000 Hz, and filtered online from DC to 250 Hz. Offline, data were referenced to the average of all scalp-recorded electrodes, band-pass filtered between 0.1 and 30 Hz (zero phase-shift Butterworth, 24 dB/octave), corrected for ocular artifacts (Gratton et al., 1983), and epoched into 700 ms segments (−200 to 500 ms). EEG segments were linear detrended, baseline corrected, and artifact rejected with a criterion of ±150 μV. Remaining EEG segments were pooled across posterior electrodes O1, Oz, O2, P7, P8, PO7, and PO8.

**Figure 10:**
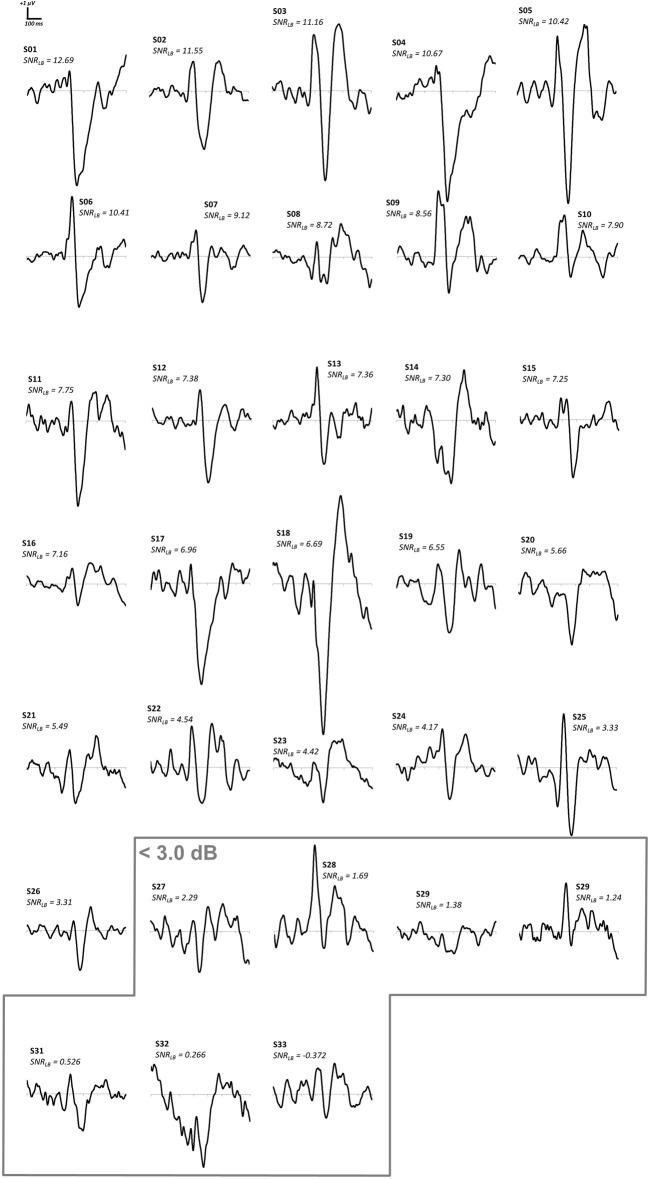
**Representative waveforms and *SNR_LB_* values for 33 subjects in an existing ERP dataset**. The seven subjects (highlighted in gray) that fail to meet an *SNR_LB_* criterion of 3.0 dB exhibit highly noisy ERP waveforms.

The number of segments used to form bootstrap ERPs, *S*, was 112 prior to artifact rejection but was determined individually for each subject as the total number of segments surviving artifact rejection procedures, *N*, divided by the number of conditions (sixteen). *SNR_LB_* was then computed for each subject using 9999 bootstraps (Figure 1). Representative ERP waveforms were derived for each subject by taking the bootstrap ERP (out of the 9999) at the median SNR value. Representative bootstrap ERPs from all 33 subjects are given in Figure 10. The lower bound of SNR-CIs for the 33 subjects ranged from −0.37 to 12.68 dB (*M* = 6.17 dB, *Mdn* = 6.96 dB, SD = 3.50 dB, *IQR* = 5.32 dB) with 7 of the 33 subjects failing to meet the recommended *SNR_LB_* criterion of 3.0 dB. Inspection of representative waveforms indicates that those seven subjects failing to meet an *SNR_LB_* criterion of 3.0 dB either failed to show a clear pattern of VEP components (S29) or had waveforms with some semblance of VEP components but were overwhelmed by noise (S27, S28, S30, S31, S32, S33). Removal of these seven subjects yielded an *SNR_LB_* range from 3.31 to 12.68 dB (*M* = 7.56 dB, *Mdn* = 7.33, SD = 2.55 dB, *IQR* = 3.83 dB). These remaining 26 subjects (with *SNR_LB_* exceeding 3.0 dB) had clearly delineated VEP components that were plainly discernable from baseline noise.

With current approaches to subject exclusion in ERP studies, it is unlikely the same seven subjects would all have been rejected from this dataset. Several of the rejected subjects exhibit relatively high amplitude P1 and N1 components of the visual evoked potential (S28, S30, S31, and S32). These subjects’ waveforms may conform to the pattern of a VEP well enough for an investigator to choose to include these subjects in the sample. However, when the presence of these components is considered in the context of their baseline noise (as indexed by *SNR_LB_*), these subjects’ waveforms should be considered unsuitable for inclusion in group-level statistics. The use of *SNR_LB_* eliminates the selection biases of the investigator and provides an objective numeric criterion of subject waveform quality.

## Discussion

An investigator’s decision to exclude ERP subjects from group-level analyses is most often determined by mere visual inspection of ERP waveforms. Thus, this critical quality control step of ERP experiments is qualitative, subjective, and lacks any clear standards or well-defined criteria. The issues of subject exclusion are pervasive in the ERP literature and there is great need for the establishment of a quantitative, objective, and standardized method for subject exclusion. Here, we provide such a method: a simple statistical measure that quantifies the quality of an ERP waveform as the statistical separation of the evoked signal from baseline noise, the SNR-CI. SNR-CIs are computed at the individual subject level using a bootstrap procedure to resample mean ERP waveforms, generate a large distribution of ERP SNR values, and determine the 90% CI of this distribution (Figure 1). The lower bound of this SNR-CI, the *SNR_LB_*, provides a minimum statistical boundary of the signal quality of an individual subject waveform, which can be evaluated against a desired SNR criterion to determine whether or not to include the subject’s data in a final sample. In addition to providing an objective criterion for subject exclusion, *SNR_LB_* can also provide a useful metric capturing the quality of individual subjects in an ERP dataset. Reporting the range, mean, and standard deviation of *SNR_LB_* can provide a subject-level summary of the quality of an ERP sample, improving data transparency and increasing reader confidence in reported results.

We took three approaches to demonstrate the efficacy and utility of employing *SNR_LB_* as a subject exclusion criterion and metric of ERP signal quality. First, we used Monte Carlo simulations on synthetic ERP subjects. We embedded a known ERP waveform in varying levels of noise to demonstrate that *SNR_LB_* could accurately classify the presence of signal averaged ERPs as well as the quality of a signal averaged waveform (assessed by statistical fit to the underlying waveform). In both cases, *SNR_LB_* exhibited exceptionally high accuracy in classifying subject waveforms over broad ranges of background noise. Moreover, these simulations demonstrated a strong monotonic relationship between *SNR_LB_* and statistical fit (*R*^2^) to the true underlying waveform: increasing values of *SNR_LB_* were associated with increasingly better fit to the underlying signal (*r_s_* = 0.973). Second, using both simulated and real ERP data, we provided qualitative visual demonstration of the relationship between varying levels of *SNR_LB_* and the form and pattern of resultant ERP waveforms in individual subjects. These demonstrations further illustrate a clear relationship between *SNR_LB_* and ERP quality: increasing values of *SNR_LB_* were associated with decreasing levels of noise. In a final demonstration of the utility of *SNR_LB_*, we applied the method to an existing ERP dataset to illustrate the poor quality of subject waveforms that failed to meet a recommended *SNR_LB_* criterion of 3.0 dB. Waveforms of such rejected subjects showed an absence of typical ERP components or substantial levels of baseline noise. Together, simulations and qualitative demonstrations strongly support *SNR_LB_* as a means of establishing subject exclusion criteria and as an indicator and metric of ERP waveform quality.

There are a number of practical issues in regards to the application of *SNR_LB_* to an empirical ERP dataset that merit further discussion:

1. *Criterion value for subject exclusion*. Our Monte Carlo simulations suggest that an *SNR_LB_* value of 3.0 dB should serve as an effective minimum criterion for the inclusion of a subject in an ERP sample. This value is intended simply as a heuristic for subject exclusion and cannot account for every situation or context. In some cases, an investigator may wish to establish a higher criterion value for greater assurance of the reliability of the subject waveforms included in a sample. Other cases may warrant a somewhat lower criterion of *SNR_LB_*, as in studies drawing samples from special populations (e.g., patients) or where forming ERPs from a large number of trials is not possible. A criterion of *SNR_LB_* should never be lower than 0 dB, as this establishes an absolute statistical minimum for the presence of an evoked response. Conversely, investigators should be cautioned not to set an unnecessarily high *SNR_LB_* criterion. An *SNR_LB_* criterion for subject exclusion is intended to eliminate subjects that fail to show a reliable level of signal strength. It is not intended to select only those subjects with extraordinarily high SNR, as doing so can artificially clip the natural variability of SNR in an ERP sample.
2. *Electrode pooling*. Computations of *SNR_LB_* on real datasets in this paper involved pooling relevant electrode positions into a single electrode, representative of the ERP (selected according to scalp distributions). In the application of *SNR_LB_* to an experimental ERP dataset, the electrodes pooled for the computation of *SNR_LB_* should be the same as those used for statistical analyses. If it is impractical or illogical to pool electrodes of interest (e.g., a component reverses polarity between electrodes of interest), then pooling should occur following computation of SNR during each bootstrap. That is, average SNR at each electrode is used to compute *SNR_LB_* as opposed to the SNR of an average electrode.
3. *Number of segments used to compute SNR_LB_*. A third issue in the application *SNR_LB_* to an ERP dataset relates to the number of segments sampled, *S*, for signal averaging each bootstrap ERP (Figure 1B). Determination of this value is critical to the accuracy of the resultant *SNR_LB_* estimates. A value of *S* that is too low will underestimate *SNR_LB_* and a value too high will overestimate *SNR_LB_*. The value of *S* selected for each subject should be equivalent to the number of segments that will be used to form condition averages after artifact rejection. For an experiment with an equal number of trials in all conditions, we recommend that the value of *S* be determined as the mean number of trials per condition that survive artifact rejection (i.e., *N* divided by the number of conditions; see Figure 1). However, many ERP experimental designs involve conditions with significant imbalances in the number of trials. In these designs, we recommend that the value of *S* be determined as the mean number of trials from those conditions with the fewest number trials. Thus, *SNR_LB_* should be determined based on the experimental manipulations with the fewest trials for signal averaging.
4. *Baseline correction requirement*. The calculation of SNR (Figure 1C) requires that the ERP is baseline corrected according to the prestimulus interval. That is, the prestimulus interval is set to have a mean of zero. If the design of an experiment does not permit ERPs to be baseline corrected or if an evoked signal is also expected in the baseline interval, special considerations must be taken establish an alternative time period to serve as a baseline interval.
5. *SNR_LB_ reporting*. In addition to serving as a criterion for subject exclusion, summary statistics of *SNR_LB_* also provide important information regarding the subject-level quality of an ERP sample. We recommend reporting the mean, median, standard deviation, inter-quartile range, minimum, and maximum of a sample’s *SNR_LB_*. Reporting these statistics of *SNR_LB_* in ERP papers can convey a significant degree of information regarding the overall quality of the individual subjects going into a sample and should be reported for the sample both before and after subject exclusion. Regardless of the choice to perform subject exclusion or the outcome of subject exclusion procedures, such summary statistics of *SNR_LB_* should still be reported so that the reader can ascertain the reliability of the ERPs used in statistical analyses and grand average waveforms.
6. *Limitations*. *SNR_LB_* provides an objective metric of ERP quality on the basis of signal vs. noise but does not provide any information regarding undesirable or anomalous patterns in the ERP. *SNR_LB_* is not sensitive to polarity or qualitative patterns, only to the strength of a post-stimulus evoked response. There are some circumstances in which a subject waveform may yield a sufficiently high value of *SNR_LB_* but may still be undesirable for inclusion in an ERP sample due to an anomalous ERP pattern. For example, a subject could have an *SNR_LB_* of 10 dB but also show an atypical polarity reversal of a canonical ERP component (e.g., an inverted visual P1). In this case, the investigator may still wish to exclude this subject as an outlier. Thus, *SNR_LB_* only provides an objective and quantitative metric for subject exclusion on the basis of SNR but cannot flag subjects with anomalous ERP waveforms.

Though our *SNR_LB_* measure provides a quantitative and objective method approach to subject exclusion, there are several alternative views and approaches for dealing with poor quality subject waveforms that should also be noted. A first alternative is to simply not perform subject exclusion on the basis of waveform quality, instead including all subjects in group-level analyses so long as there was no experimental error during the acquisition of the subject’s dataset (e.g., high electrode impedance). However, including evoked responses that fail to overcome baseline levels of noise will have a deleterious effect on experimental results as this is equivalent to a measurement error. An evoked response is, by definition, an increase in post-event signal strength relative to a pre-stimulus baseline. If a post-event signal is no stronger than its baseline then no evoked response is objectively present and a hypothesis cannot truly be evaluated. There are many factors that can cause evoked response to fail to emerge above the noise, some external (e.g., high electrode impedance) and some internal (e.g., subject fatigue). Many of these factors cannot be easily observed, measured, or quantified, but nonetheless negatively impact the ERP signal. Unless other precautions are taken, the inclusion of subjects with poor SNR can have a significant negative impact on hypothesis testing. The *SNR_LB_* measure provides a statistical boundary that can be used to determine when a true evoked response has been measured in a subject. A second alternative approach to subject exclusion is to employ robust statistics (Wilcox, 2012) rather than strictly identifying and removing subjects with poor quality waveforms (as with *SNR_LB_* criteria). The application of robust statistical methods (Wilcox and Keselman, 2003; Keselman et al., 2008; Wilcox, 2012) to group-level ERP analyses can mitigate the impact of outlier subjects’ ERPs on the outcome of a hypothesis test (Rousselet and Pernet, 2011), and are regularly used in the ERP literature (e.g., Dien et al., 2006; Franklin et al., 2007; Rousselet et al., 2008; Clawson et al., 2013; Desjardins and Segalowitz, 2013). Though it is advantageous to exclude subject waveforms that fail to exhibit clear evoked responses, robust statistics form a viable alternative (or complementary approach) to subject exclusion procedures. Several software packages are freely available for performing robust statistics on ERP datasets (Maris and Oostenveld, 2007; Dien, 2010; Litvak et al., 2011; Pernet et al., 2011). A final alternative to setting an absolute rejection threshold based on *SNR_LB_* is to instead derive a measure of SNR from each subject’s grand average waveform and apply a standard outlier rejection procedure to identify those subjects with unusually low SNR. For example, an iterative outlier rejection procedure could be run on the SNR of an ERP sample, rejecting subjects having an SNR more than two standard deviations below the group mean. Though such an approach is a valid method of identifying outlier subjects with poor signal quality, deriving a measure of SNR from a grand average waveform overinflates the condition level SNR. In this case, we would suggest deriving a mean SNR from a bootstrap of all EEG segments using a value of *S* equivalent to the mean number of segments per condition (Figure 1).

In summary, we describe a quantitative measure of ERP signal quality based on the bootstrap computation of SNR-CIs in individual subjects. The lower bound of these SNR-CIs, the *SNR_LB_*, provides a standardized and objective criterion to exclude poor-quality subjects from ERP samples. *SNR_LB_* can also be reported in ERP papers as a summary statistic to convey the quality of individual subject waveforms of the ERP experiment. Though we only describe the computation and application of bootstrap SNR-CIs as they relate to ERP studies, the approach may also be readily applied to any event-related physiological data (e.g., evoked magnetic field, local field potential, motor-evoked potential, startle response, post-auricular reflex, and skin conductance response). SNR-CIs may also be further adapted to extend to evoked frequency-domain signals such as event-related synchronization/desynchronization and steady-state evoked potentials. The code for computing estimates of *SNR_LB_* is freely available for download at http://www.uark.edu/ua/parkslab/SNRLB and https://figshare.com/s/f6da4150953b0f9cc3bd.

## Author Contributions

NAP and MAG conceived of and implemented the described methodology. MAG, SML and MEY collected/analyzed data and conducted literature reviews.

## Conflict of Interest Statement

The authors declare that the research was conducted in the absence of any commercial or financial relationships that could be construed as a potential conflict of interest.

## Footnotes

^1^The SNR of an ERP may also be calculated as the mean SNR of individual segments rather than SNR of the averaged waveform. Deriving SNR before signal averaging yields a measure of SNR that reflects all post-event activity rather than only the phase-locked response (the ERP). As such, calculation of SNR on signal averages is the most accurate representation of ERP quality.

^2^A criterion of 6.0 dB was the upper limit achievable across these four subjects given the chosen parameters.

^3^A criterion of 10.0 dB was the upper limit achievable across these four subjects given the chosen parameters.
